# Mitigation of soil N_2_O emission by inoculation with a mixed culture of indigenous *Bradyrhizobium diazoefficiens*

**DOI:** 10.1038/srep32869

**Published:** 2016-09-16

**Authors:** Hiroko Akiyama, Yuko Takada Hoshino, Manabu Itakura, Yumi Shimomura, Yong Wang, Akinori Yamamoto, Kanako Tago, Yasuhiro Nakajima, Kiwamu Minamisawa, Masahito Hayatsu

**Affiliations:** 1 Institute for Agro-Environmental Sciences, National Agriculture and Food Research Organization (NARO), 3-1-3, Kannondai, Tsukuba, Ibaraki 305-8604, Japan; 2 Graduate School of Life Sciences, Tohoku University, 2-1-1 Katahira, Aoba-ku, Sendai, Miyagi 980-8577, Japan; 3 Advanced Analysis Center, NARO, 3-1-3, Kannondai, Tsukuba, Ibaraki 305-8604, Japan

## Abstract

Agricultural soil is the largest source of nitrous oxide (N_2_O), a greenhouse gas. Soybean is an important leguminous crop worldwide. Soybean hosts symbiotic nitrogen-fixing soil bacteria (rhizobia) in root nodules. In soybean ecosystems, N_2_O emissions often increase during decomposition of the root nodules. Our previous study showed that N_2_O reductase can be used to mitigate N_2_O emission from soybean fields during nodule decomposition by inoculation with *nosZ*++ strains [mutants with increased N_2_O reductase (N_2_OR) activity] of *Bradyrhizobium diazoefficiens*. Here, we show that N_2_O emission can be reduced at the field scale by inoculation with a mixed culture of indigenous *nosZ*+ strains of *B. diazoefficiens* USDA110 group isolated from Japanese agricultural fields. Our results also suggested that nodule nitrogen is the main source of N_2_O production during nodule decomposition. Isolating *nosZ*+ strains from local soybean fields would be more applicable and feasible for many soybean-producing countries than generating mutants.

Agricultural soil is the single largest source of global anthropogenic nitrous oxide (N_2_O) emission^1^, accounting for approximately 59% of anthropogenic emissions^2^. N_2_O is a greenhouse gas that is also detrimental to the ozone layer^2^. The global warming potential of N_2_O is ~300-fold higher than that of CO_2_ on a molar basis, and the concentration of N_2_O has increased at a rate of 0.73 ppb yr^−1^ over the last three decades^2^.

Soybean (*Glycine max* [L.] Merr.) is one of the most important crops in the world. Soybean is grown on 6% of the world’s arable land, and its production has dramatically increased from 26 Mt in 1961 to 308 Mt in 2014 (ref. 3). The soybean production area is expected to increase more than that of other crops^4^. As a leguminous crop, soybean hosts symbiotic nitrogen-fixing soil bacteria (rhizobia) that can also produce N_2_O in root nodules^5^. In soybean ecosystems, increase of N_2_O emission during decomposition of the root nodules has often been reported^6^. Organic nitrogen inside the decomposing nodules is mineralized to NH_4_^+^ followed by nitrification and denitrification that produce N_2_O (Fig. 1a)^7^,^8^. N_2_O is then emitted into the atmosphere or is further reduced to N_2_ by N_2_O reductase (N_2_OR), which is encoded by the *nosZ* gene. *Bradyrhizobium diazoefficiens* is a nitrogen-fixing rhizobium that also possesses a denitrification pathway^8^. Both *B. diazoefficiens nosZ*+ (strains that have the *nosZ* gene) and *nosZ*− (strains that do not have the *nosZ* gene) strains are found in soil^9^. Denitrification by *nosZ*− strains produces N_2_O because they lack *nosZ*, whereas *nosZ*+ strains can reduce N_2_O to N_2_ (Fig 1a).

Enhancing microbial N_2_OR activity has been suggested as an N_2_O mitigation option^10^, and mitigation of N_2_O using *nosZ* has been demonstrated on the laboratory scale. Pure culture and vermiculite pot experiments showed lower N_2_O emission by *nosZ*+ strains^11^ and *nosZ*++ strains (mutants with increased N_2_OR activity)^12^,^13^ of *B. diazoefficiens* than by *nosZ*− strains. A pot experiment using soil confirmed these results^14^. In addition to the use of *B. diazoefficiens*, transgenic plants expressing N_2_OR following introduction of soil bacterial *nosZ* were generated to reduce N_2_O emission^15^. However, no field-scale study has been reported except our previous study, which showed that N_2_OR can be used to mitigate N_2_O emission by inoculation with *nosZ*++ strains of *B. diazoefficiens*^13^. Although it is an effective approach, generating *nosZ*++ mutants requires time, cost, and technical skills, and the field use of genetically modified microbes is regulated in many countries. In contrast, isolating indigenous strains from field soil is easy and cost-effective in comparison with generating mutants. Moreover, isolated indigenous strains may be more competitive than mutants with native field strains. Here we report the mitigation of N_2_O emission from a soybean field by inoculation with a mixed culture of indigenous *nosZ*+ strains of *B. diazoefficiens* isolated from agricultural fields, without the use of a mutant (Fig. 1b).

## Results and Discussion

### Construction of cell mixture of indigenous USDA110 group isolates

**Figure 1:**
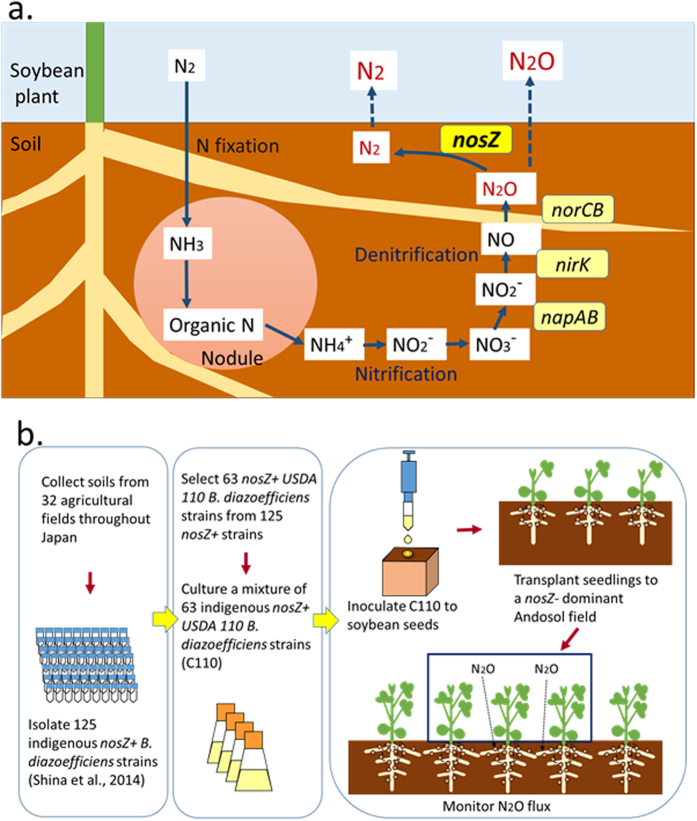
(**a**) Microbial pathway involved in N_2_O production from decomposition of root nodules. During decomposition of nodules, nitrogen becomes available for soil microorganisms. Using this nitrogen, *B. diazoefficiens nosZ*+ strains sequentially reduce nitrogen oxides during denitrification (NO_3_^−^ → NO_2_^−^ → NO → N_2_O → N_2_), with each step catalyzed by specific reductases encoded by denitrifying genes: *napA* (periplasmic nitrate reductase), *nirK* (copper-containing nitrite reductase), *norCB* (nitric oxide reductase), and *nosZ* (nitrous oxide reductase), respectively. However, denitrification by *B. diazoefficiens nosZ*− strains produces N_2_O because they lack the *nosZ* gene that encodes N_2_O reductase (N_2_OR). Both *nosZ*+ and *nosZ*− strains are found in the soil. (**b**) Design of the experiment. First, soils were collected from 32 agricultural fields throughout Japan. Then, 125 indigenous *nosZ*+ *B. diazoefficiens* strains were isolated (Shina *et al.*)^9^. From these 125 strains, 63 indigenous *nosZ*+ strains of *B. diazoefficiens* USDA110 group were selected (C110), because nitrogen fixation in USDA110 is higher than that in other strains (Itakura *et al.*)^18^. C110 was cultured and inoculated onto soybean seeds in biodegradable pots. For control plots, soybean seeds were inoculated with soil from the experimental field. Soybean seedlings were grown for 10 days in a greenhouse and then transplanted into a *nosZ-* dominant Andosol field. Annual N_2_O flux was monitored and mitigation of N_2_O production by soybean nodules of inoculated strains was evaluated.

Although (brady)rhizobia have been used as inoculants for legume crop production worldwide, rhizobial inoculation is often ineffective in the presence of indigenous rhizobia in soils because of the problem of so-called competition between inoculants and (brady)rhizobial populations indigenous to field soils^16^,^17^. Many genomic variations have been found even in isolates in a *B. deazoefficiens* collection^9^,^18^ (Itakura *et al.* unpublished results), suggesting that field inoculation with a mixture of *B. deazoefficiens* isolates could overcome the competition problem. We accordingly prepared a cell mixture (C110) of native *B. diazoefficiens* as an inoculant (Table S1). Shiina *et al.*^9^ isolated 125 native *nosZ*+ *B. diazoefficiens* from 32 field soils in Japan. Because *B. diazoefficiens* strains belonging to the USDA110 group showed high ability to fix N_2_ in soybean nodules^18^,^19^, we selected 63 of the 125 isolates whose 16S–23S rRNA ITS sequences were identical to that of strain USDA110 (Table S1). The cell mixture C110 derived from these 63 isolates was used in a field experiment as an inoculant (Fig. 1b). We expected that field inoculation efficiency could be increased if more-competitive isolates were included in C110.

### Inoculation efficiency and gene expression in the field experiment

We conducted a two-year field experiment to test the effectiveness of C110 inoculation in reducing N_2_O emission in an Andosol field dominated by *nosZ*− strains. In our previous study, postharvest N_2_O emission was significantly reduced by *nosZ*++ (mutants with increased N_2_OR activity) inoculation, whereas the proportion of *nosZ*++ nodules in the field experiment was only 23% (ref. 13). We expected that increasing the proportion of inoculated strains in nodules might reduce more N_2_O from decomposition of nodules. In addition to the construction of C110, we improved our germination and inoculation methods to increase the proportion of inoculated strains of nodules. To increase the proportion of cotyledon emergence, soybean seeds were germinated in trays filled with moist vermiculite for one day instead of being seeded in soil-filled pots as in our previous study^13^. With this change, the proportion of cotyledon emergence increased from approximately 30% for soil to 95% for vermiculite germination. The low proportion of cotyledon emergence for soil may have been observed because maintaining optimal soil water content for germination is much more difficult for small and water-permeable biodegradable pots filled with soil than for large trays filled with vermiculite. After soybean seeds were germinated in moist vermiculite for a day, they were transferred to biodegradable pots filled with soil. For the pots, soil were collected from a nearby Andosol orchard that showed a lower *nod C* copy number than the Andosol soil of the experimental field (Fig. S1), instead of using soil from the experimental field as in Itakura *et al.*^13^. Immediately after transfer to the pots, the seeds were inoculated with the mixed culture C110 (*nosZ*+) or soil from the experimental field (native). The seedlings were grown for 10 days in a greenhouse and then were transplanted to the Andosol field. As a result of these changes in methods, the proportion of *nosZ*+ nodules in *nosZ*+ inoculated plots in this study were 71.4% to 82.8% from August to October (Table 1), much higher than the 23% for inoculated strain in our previous study^13^. Our results also showed that the proportion of *nosZ*+ nodules remained high from vegetative to full maturity stage in *nosZ*+ inoculated plots (Table 1). Furthermore, the proportion of *nosZ*+ outside pots in *nosZ*+ inoculated plots were 38–68%, significantly higher than that in native plots (*P* < 0.001) on all sampling dates. This result indicated that C110 was able to infect soybean roots outside of pots where native rhizobia populations were high. In addition, C110 was more competitive with native strains than *nosZ*++, which showed 0% of inoculated strain outside of the pots in our previous field experiment^13^.

Gene expression analysis showed that *nosZ* expression was significantly higher in nodules collected from *nosZ*+ inoculated plots than in those from native plots (Table 2), suggesting that N_2_OR activity in *nosZ*+ inoculated plots was higher than that in native plots. In contrast, no significant difference in *nirK* expression between the two treatments was found (Table 2), suggesting that the denitrification process before N_2_O reduction did not differ between the treatments.

### N_2_O emissions in the field experiment

Nodule decomposition begins during the late growth period^7^. N_2_O fluxes increased after fertilizer application and the nodule decomposition period (end of August to mid-November) in 2013 (Fig. S2) and in 2014 (Fig. S3). In some studies, nodule decomposition and the consequent N_2_O emission were observed from late growth period until after harvest^13^,^20^. N_2_O emissions during the nodule decomposition period were larger than those after fertilizer application in both years. N_2_O fluxes from the *nosZ*+ inoculated plots were lower than those from native plots during the nodule decomposition period in both years. Consequently, cumulative N_2_O emission during the nodule decomposition period in *nosZ*+ inoculated plots was significantly lower than that of native plots based on a mixed linear model using two years of field data (Table 3; *P* < *0*.*05*). In this study, significant mitigation of N_2_O by *nosZ*+ inoculation was observed during nodule decomposition period; that is, before and after harvest, whereas only postharvest N_2_O emission showed a significant decrease following *nosZ*++ inoculation in our previous study^13^.

Increased N_2_O emission in soybean ecosystems during the harvest period has been reported^13^,^20^,^21^,^22^. Uchida and Akiyama^6^ reviewed N_2_O emissions from soybean fields and reported that 0–13.4% of soybean residual N was emitted as N_2_O after harvest (average: 1.3% ± 2.7%) in previous studies. Although cumulative N_2_O emissions in our field experiments were relatively low, N_2_O emission from a soybean field during nodule decomposition can reach as high as 5 kg N ha^−1^ (ref. 22).

### N_2_O production rates in the field experiment

**Figure 2:**
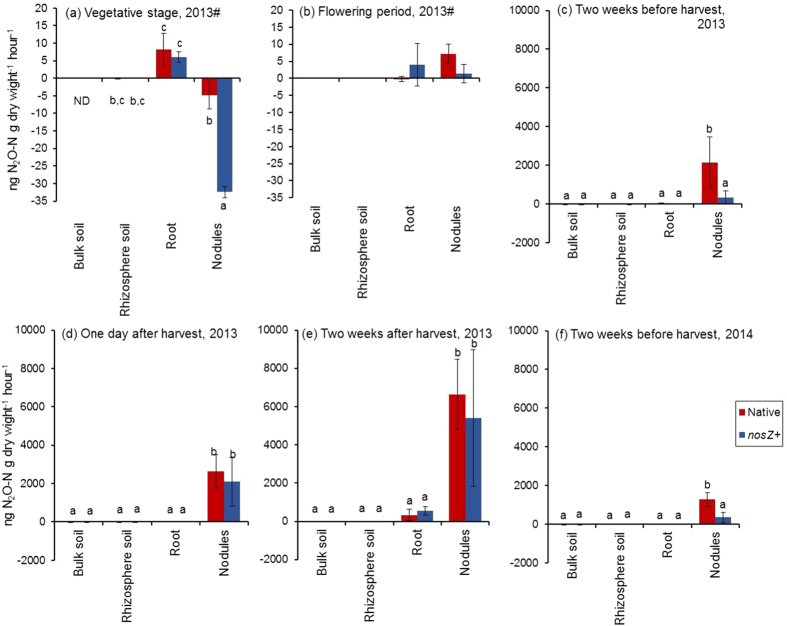
N_2_O production rates from bulk soil, rhizosphere soil, and root and nodule samples collected from the experimental field at different growth stages in 2013 and 2014. (**a**) Vegetative stage (five weeks after inoculation, July 29, 2013), (**b**) flowering period (seven weeks after inoculation, August 12, 2013), (**c**) two weeks before harvest (October 3, 2013), (**d**) one day after harvest (October 18, 2013), (**e**) two weeks after harvest (October, 29 2013), and (**f**) two weeks before harvest (October 1, 2014). Soybean seeds were inoculated at sowing with a mixed culture of *B. diazoefficiens* strains (*nosZ*+) or native (*nosZ*− dominant) (n = 3# or 5, see text).

N_2_O production rates from soil, root, and nodule samples collected from the experimental field were measured at different growth stages in 2013 (Fig. 2a–e). At the vegetative stage, N_2_O was absorbed by nodules in both treatments, and the N_2_O uptake rate was significantly higher in the *nosZ*+ treatment than in the native treatment (Fig. 2a; *P* < *0*.*05*). Sameshima-Saito *et al.*^11^ also reported N_2_O uptake by nodules from USDA110 (*nosZ*+)-inoculated plants, but no N_2_O uptake by nodules from *nosZ*−mutant-inoculated plants at the vegetative stage. Nodule N_2_O production rates increased dramatically during the nodule decomposition period (Fig. 2c,d,e), and that in the *nosZ*+ treatment was significantly lower than that in the native treatment in the two weeks before harvest (Fig. 2c; *P* < *0*.*05*), whereas no differences were found in other periods (Fig. 2d,e). In contrast, N_2_O production rates of bulk soil, rhizosphere soil, and root remained low in all growth stages (Fig. 2a–e). In 2014, N_2_O production rates were measured two weeks before harvest, and the results confirmed that nodule N_2_O production rates were much higher than those of bulk soil, rhizosphere soil, and roots (Fig. 2f). As in 2013, the nodule N_2_O production rate of the *nosZ*+ treatment in 2014 was significantly lower than that of the native treatment two weeks before harvest (*P* < *0*.*05*). These results suggested that nodules were the main source of N_2_O emission from the soybean field during the nodule decomposition period. Although our previous study^13^ also suggested the importance of nodules as a N_2_O source, N_2_O production rates from soil and nodules were not measured in that study. Moreover, a lower nodule N_2_O production rate from *nosZ*+ treatment than from the native treatment two weeks before harvest (Fig. 2d,f) suggested that the field-scale reduction of N_2_O emission in the *nosZ*+ plot (Table 3) was due to a lower N_2_O production rate from the *nosZ*+ nodules.

### Soil and nodule inorganic N contents in the field experiment

**Figure 3:**
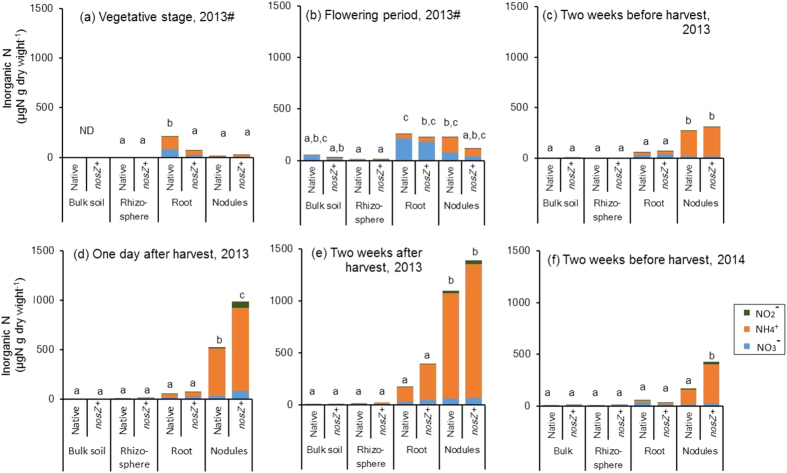
Inorganic N content in bulk soil, rhizosphere soil, and root and nodule samples collected from the experimental field at different growth stages in 2013 and 2014. (**a**) Vegetative stage (five weeks after inoculation, July 29, 2013), (**b**) flowering period (seven weeks after inoculation, August, 12 2013), (**c**) two weeks before harvest (October 3, 2013), (**d**) one day after harvest (October 18, 2013), (**e**) two weeks after harvest (October 29, 2013), and (**f**) two weeks before harvest (October 1, 2014). Soybean seeds were inoculated at sowing with a mixed culture of *B. diazoefficiens* strains (*nosZ*+) or native (*nosZ*− dominant) (n = 3# or 5, see text).

Soil and nodule inorganic N contents also suggested that nodules were the main N source of N_2_O emission during the nodule decomposition period in the soybean field. Nodule inorganic N content, mostly NH_4_^+^, remained low from the vegetative stage to flowering (Fig. 3a,b). It began to increase two weeks before harvest (Fig. 3c), and then dramatically increased just before harvest and two weeks after harvest in 2013 (Fig. 3d,e). In contrast, inorganic N content in bulk soil, rhizosphere soil, and roots remained low in all periods (Fig. 3a–f). As in 2013, nodule inorganic N, mainly NH_4_^+^ content, was higher than those of bulk soil, rhizosphere soil, and roots at two weeks before harvest in 2014 (Fig. 3f).

Seasonal changes in bulk soil NO_3_^−^ and NH_4_^+^ concentrations showed that NH_4_^+^ increased just after fertilizer application and consequent increase in NO_3_^−^ by nitrification (Figs S4 and S5). However, bulk soil inorganic nitrogen concentrations did not increase during the nodule decomposition period and did not differ significantly among treatments in either year, a finding similar to that in our previous study^13^. These results suggested that nodules were the main N source for N_2_O emission rather than nitrification and denitrification of soil nitrogen during the nodule decomposition. The nodule N_2_O production rate (Fig. 2) also suggested that nodules were the main N source for N_2_O emission during nodule decomposition. Inaba *et al.*^8^ reported that N_2_O emitted during nodule decomposition in a pot experiment was derived from fixed nitrogen in the nodules. They also reported that *B. diazoefficiens nosZ*+ strains reduced both N_2_O produced by *B. diazoefficiens* and N_2_O produced by other soil microorganisms during nodule decomposition. Although soybean nodules have been proposed as the main N source for N_2_O emission during nodule decomposition^7^,^8^,^13^,^20^, the present study is the first to provide evidence that nodule inorganic N content and N_2_O production rate of nodules increased with N_2_O flux during nodule decomposition at the field scale.

## Conclusion

In our previous report^13^, we showed that inoculation with the *nosZ*++ strain of *B. diazoefficiens* significantly decreased postharvest N_2_O emission. The *nosZ*++ strain used in the field study was a genetically unmodified mutant generated using a proofreading-deficient technique^12^. Although it was an effective approach for reducing N_2_O emissions from soybean fields, generating *nosZ*++ mutants requires more time, cost, and technical skill than isolating indigenous *nosZ*+ strains from soil as in the present study. Also, inoculation of soybean with indigenous strains has a long history and has been practiced commercially in many countries^23^, whereas use of mutants, especially genetically modified mutants, may need to receive public acceptance before commercial use. In addition, we used a mixed culture of 63 *nosZ*+ strains of USDA 110 group from agricultural fields from Japan, rather than selecting one strain from the *nosZ*+ collection. The mixture of many strains provides more diversity and is accordingly expected to be more competitive than a single strain with native strains and also more adaptable to various environments and robust to extreme weather, such as drought, heat, and heavy rainfall. Moreover, using *nosZ*+ strains isolated from local agricultural fields would have little effect on the ecosystem. Thus, isolating *nosZ*+ strains from local soybean fields would be more applicable and feasible for many soybean-producing countries than generating mutants.

Crop production needs to increase by approximately 60–100% from 2007 to 2050 to meet global food demand^24^. The increasing demand for food and biofuel is likely to require increasing N inputs even further, although anthropogenic reactive N input into the biosphere has already exceeded a proposed planetary limit^24^. Consequently, N_2_O emission from agriculture is likely to continue to increase^25^. To reduce N_2_O emission from agricultural soils, many mitigation options have been proposed, but very few options are available^26^: they include nitrification inhibitors, polymer-coated fertilizers^27^, and reducing the input of anthropogenic reactive nitrogen^28^. No biological method had been demonstrated in the field before our previous study^13^. The biological approach to reduce N_2_O emission is still in an early stage of development, but the present study showed that inoculation with indigenous *nosZ*+ strains has high potential to mitigate N_2_O emission from soybean ecosystems without the use of mutants. This approach can also be applied to other leguminous crops. Inoculation of alfalfa with the endosymbiont *Ensifer meliloti* carrying the *nosZ* gene was recently suggested as a potential mitigation option^29^. Furthermore, there is potential to mitigate N_2_O emission by using the *nosZ* gene in various other soil microbes^30^.

## Methods

### Bacterial strains, media, and construction of cell mixture

A cell mixture named C110 was prepared from 63 isolates belonging to a USDA110 group of *Bradyrhizobium diazoefficiens* that were collected from soybean nodules in 32 agricultural fields of Japan^9^ (Table S1). The bradyrhizobial isolates were grown individually for five days at 30 °C in HM broth medium^31^ supplemented with 0.1% L-arabinose (w/v) and 0.025% (w/v) yeast extract. The turbidities of the cultures were adjusted to OD_660_ = 1 with HM broth medium, and the cultures were mixed in equal amounts. One milliliter of the cell mixture was inoculated into fresh HM broth medium (100 ml) and cultured at 30 °C for five days. The resulting C110 was grown at 30 °C in modified AG medium^32^ supplemented with 0.3% (w/v) arabinose, 0.3% (w/v) yeast extract, and 0.3% (w/v) sodium gluconate for field inoculation.

### Field experiment

The experimental field was located at the Institute for Agro-Environmental Sciences, Japan (36°01′N, 140°07′E). The Andosol field (*nosZ*−, 98%; *nosZ*+, 2%) was divided into 6 × 6 m plots. The treatments were inoculation with a mixture of 63 *nosZ*+ strains of USDA110 group (C110; *nosZ*+) or with native rhizobia (native) (five replicates of field plots with blocked random design).

To increase the proportion of cotyledon emergence, soybean seeds were germinated for one day in trays of moist vermiculite. Then soybean (*Glycine max* [L.] Merr., ver. Tachinagaha) seeds were planted in biodegradable Jiffy pots (Jiffy International AS, Kristiansand, Norway) filled with Andosol soil collected from an orchard located approximately 100 m from the experimental field. The orchard soil was chosen because it had a lower population of native soybean bradyrhizobia than, but soil properties similar to those of, the experimental field. Fruit trees had been grown in the orchard for more than 40 years, and thus had experienced no soybean cultivation for at least 40 years. Soybean seeds were inoculated with C110 (*nosZ*+) or 50 ml of soil from the experimental field (native) on June 26, 2013 and June 18, 2014. Soybean seedlings were then grown in a greenhouse under natural light and then transplanted into the field on July 3, 2013 and June 25, 2014. Basal fertilizer was applied as a compound fertilizer (30 kg N ha^–1^) one day before transplanting the soybean seedlings. Soybean crops were harvested on October 17, 2013 and October 10, 2014, aboveground residues were removed, and only roots and stubble were left in the field. N_2_O emission was measured every two to four days using an automated gas sampling system^33^. N_2_O concentrations were determined on a gas chromatograph equipped with an electron capture detector (GC-ECD). The effect of *nosZ*+ on N_2_O emission based on data from two years of field experiments was evaluated using a mixed linear model.

### N_2_O production rates of soil, roots, and nodules

Bulk soil, rhizosphere soil, and root and nodule samples were collected from the experimental field at five different growth stages in 2013. Samples were also collected two weeks before harvest in 2014 to confirm the results of 2013. N_2_O production rates of these samples were determined in an incubation experiment. Bulk soil was randomly collected from five points (0 to 5 cm) in each plot and mixed in a plastic bag to produce a composite sample. Root segments growing inside the Jiffy pot were collected along with rhizosphere soil. Field samples were immediately transferred to the laboratory. There, root samples were separated into rhizosphere soil, roots, and nodules. Bulk soil, rhizosphere soil, and root and nodule samples were transferred to glass vials. These were sealed with butyl rubber stoppers and incubated at 25 °C for 30 min. The nodule incubation experiments were started one hour after the field sampling to reflect N_2_O production rate in the field. Because our pre-experiment results showed that N_2_O production rate of nodules decline with time after sampling, it was important to incubate nodules as soon as possible after the field sampling, but 1 h was needed for transportation of samples and nodule sample preparation. The root and soil incubation experiments were also performed simultaneously. Gas samples were collected from vials 0, 15, 30 min after sealing. N_2_O concentrations of the gas samples were determined with the GC-ECD.

Details of all methods are provided in the Supplementary Information.

## Additional Information

**How to cite this article**: Akiyama, H. *et al.* Mitigation of soil N_2_O emission by inoculation with a mixed culture of indigenous *Bradyrhizobium diazoefficiens. Sci. Rep.* **6**, 32869; doi: 10.1038/srep32869 (2016).

## Supplementary Material

Supplementary Information

## Figures and Tables

**Table 1:** Nodule number and nodule occupancy in the field experiment in 2013 and 2014.

Sampling date	Treatment	Nodule number (plant^−1^)			Nodule occupancy by *nosZ*+ (%)		
		Inner	Outer	Total	Inner	Outer	Total
2013							
July 23	**Native**	47 ± 9	NA	47 ± 9	2.4 ± 5.4	NA	2.4 ± 5.4
	***nosZ*****+**	32 ± 3	NA	32 ± 3	89.1 ± 6.7	NA	89.1 ± 6.7
	*Statistical significance*	*P* < *0*.*05*		*P* < *0*.*05*	*P* < *0*.*001*		*P* < *0*.*001*
August 7	**Native**	67 ± 18	17 ± 5	84 ± 20	4.9 ± 6.7	6.7 ± 10.1	5.1 ± 5.5
	***nosZ*****+**	56 ± 29	29 ± 11	84 ± 16	89.4 ± 13.8	38.3 ± 22.1	71.4 ± 14.5
	*Statistical significance*	ns	*P* < *0*.*001*	ns	*P* < *0*.*001*	*P* < *0*.*001*	*P* < *0*.*001*
October 1	**Native**	75 ± 31	32 ± 13	108 ± 41	1.7 ± 6.2	7.5 ± 13.5	3.1 ± 4.8
	***nosZ*****+**	165 ± 52	39 ± 31	204 ± 71	86.5 ± 16.2	65.0 ± 18.4	82.8 ± 13.4
	*Statistical significance*	*P* < *0*.*001*	ns	*P* < *0*.*001*	*P* < *0*.*001*	*P* < *0*.*001*	*P* < *0*.*001*
2014							
August 19	**Native**	92 ± 14	66 ± 26	159 ± 31	26.6 ± 16.1	12.5 ± 15.1	21.0 ± 11.4
	***nosZ*****+**	113 ± 38	49 ± 25	162 ± 56	78.8 ± 15.5	68.3 ± 13.6	75.7 ± 13.6
	*Statistical significance*	ns	ns	ns	*P* < *0*.*001*	*P* < *0*.*001*	*P* < *0*.*001*
October 1#	**Native**	68 ± 32	44 ± 32	113 ± 57	12.5 ± 12.5	6.3 ± 10.1	10.8 ± 10.1
	***nosZ*****+**	102 ± 23	36 ± 14	138 ± 30	82.5 ± 15.0	63.8 ± 17.2	78.3 ± 11.9
	*Statistical significance*	*P* < *0*.*05*	ns	ns	*P* < *0*.*001*	*P* < *0*.*001*	*P* < *0*.*001*

Soybean seeds were inoculated with a mixed culture of 63 *Bradyrhizobium diazoefficiens* strains C110 (*nosZ*+) or native strains (Native; *nosZ*− dominant). “Inner” describes nodules on parts of roots inside the pots; “outer” describes nodules on parts of roots that extended outside the pots. Values are means ± SD (n = 15 or 10#).

Statistical significance was tested using the t-test (two-sided).

NA: data were not available because nodules were collected only from inside of pots. ns: not significant.

**Table 2:** Expression of *nirK*, *nosZ*, and *sigA* genes in soybean nodules in the field experiment in 2013 were quantified by RT-real time PCR.

Sampling day	Treatment	*nirK*	*nosZ*	*sigA*	*nirK* expression	*nosZ* expression
		(copy number g dry nodule weight^−1^)			(*nirK*/*sigA*)	(*nosZ*/*sigA*)
2013						
July 29	**Native**	2.3 × 10^8^ ± 2.1 × 10^8^	3.0 × 10^6^ ± 2.8 × 10^6^	9.5 × 10^7^ ± 6.2 × 10^7^	3.16 ± 1.62	0.03 ± 0.02
	***nosZ*****+**	4.3 × 10^7^ ± 2.2 × 10^7^	1.6 × 10^8^ ± 3.8 × 10^7^	1.2 × 10^8^ ± 3.2 × 10^7^	0.43 ± 0.27	1.45 ± 0.6
	*Statistical significance*	ns	*P* < *0*.*001*	ns	ns	*P* < *0*.*05*
August 12	**Native**	2.6 × 10^9^ ± 1.7 × 10^9^	3.7 × 10^6^ ± 2.0 × 10^6^	2.4 × 10^8^ ± 1.4 × 10^8^	12.81 ± 5.40	0.02 ± 0.00
	***nosZ*****+**	1.7 × 10^9^ ± 3.0 × 10^8^	4.0 × 10^8^ ± 9.5 × 10^7^	4.3 × 10^8^ ± 1.5 × 10^8^	4.31 ± 1.00	1.00 ± 0.27
	*Statistical significance*	ns	*P* < *0*.*001*	ns	ns	*P* < *0*.*001*
October 3#	**Native**	4.9 × 10^8^ ± 4.3 × 10^8^	1.1 × 10^6^ ± 1.0 × 10^6^	5.9 × 10^7^ ± 5.0 × 10^7^	7.12 ± 5.37	0.12 ± 0.18
	***nosZ*****+**	2.3 × 10^8^ ± 1.6 × 10^8^	2.7 × 10^8^ ± 2.0 × 10^8^	1.0 × 10^8^ ± 9.9 × 10^7^	5.22 ± 4.38	4.27 ± 2.41
	*Statistical significance*	ns	*P* < *0*.*05*	ns	ns	*P* < *0*.*05*

Soybean seeds were inoculated with a mixed culture of 63 *Bradyrhizobium diazoefficiens* strains C110 (*nosZ*+) or native strains (Native; *nosZ*− dominant). In quantification of *nosZ* mRNA, some samples showed values below the minimum limit of determination by real time PCR. These values were assigned the copy number corresponding to the minimum limit of determination when the averages were calculated. Values are means ± SD (n = 3 or 5#).

Statistical significance was tested using the t-test (two-sided).

**Table 3:** Cumulative N_2_O emission in the field experiment.

	Annual N_2_O emission	Nodule decomposition period N_2_O emission	Reduction rate
	(kgN ha^−1^)	(kgN ha^−1^)	(%)
**2013**	(from March 18, 2013 to March 17, 2014)	(from Aug 29 to Nov 15, 2013)	
**Native**	0.287 ± 0.104	0.180 ± 0.076	—
***nosZ*****+**	0.260 ± 0.115	0.130 ± 0.045	28
**2014**	(from March 3, 2014 to March 2, 2015)	(from Aug 29 to Nov 15, 2014)	
**Native**	0.246 ± 0.078	0.121 ± 0.034	—
***nosZ*****+**	0.235 ± 0.098	0.084 ± 0.033	30
Statistical significance*	ns	*P* < *0*.*05*	

Soybean seeds were inoculated at sowing with a mixed culture of *B. diazoefficiens* strains C110 (*nosZ*+) or native strains (Native; *nosZ*− dominant).

^*^Statistical significance for N_2_O emission was tested using a mixed linear model based on two years of field data.
