# Supplementary material for: Mitigation of soil N2O emission by inoculation with a mixed culture of indigenous Bradyrhizobium diazoefficiens
Source: Sci Rep. 2016 Sep 16;6:32869. doi: 10.1038/srep32869 (PMC5025649; doi:10.1038/srep32869)
Supplement: Supplementary Information [file srep32869-s1.pdf]

Supplementary information

Mitigation of soil N_2O emission by inoculation with a mixed culture of indigenous *Bradyrhizobium diazoefficiens*

Hiroko Akiyama[1], Yuko Takada Hoshino[1], Manabu Itakura[2,3], Yumi Shimomura[1,4], Yong Wang[1], Akinori Yamamoto[1,5], Kanako Tago[1], Yasuhiro Nakajima[1,6], Kiwamu Minamisawa[2]*, and Masahito Hayatsu[1]*

The following authors contributed equally to this work:
Hiroko Akiyama, Yuko Takada Hoshino and Manabu Itakura

1. Institute for Agro-Environmental Sciences, National Agriculture and Food Research Organization (NARO), 3-1-3, Kannondai, Tsukuba, Ibaraki 305-8604, Japan
2. Graduate School of Life Sciences, Tohoku University, 2-1-1 Katahira, Aoba-ku, Sendai, Miyagi 980-8577, Japan
3. Present address: Center for Ecological Evolutionary Developmental Biology, Kyoto Sangyo University, Motoyama, Kamigamo, Kita-Ku, Kyoto, 603-8555, Japan
4. Present address: Kyodo Milk Industry Co., Ltd, 20-1, Hirai, Hinode, Nishitama, Tokyo, 190-0182
5. Present address: Natural Science Research Unit, Tokyo Gakugei University, 4-1-1 Nukuikitamachi, Koganei, Tokyo, 184-8501, Japan
6. Advanced Analysis Center, NARO, 3-1-3, Kannondai, Tsukuba, Ibaraki 305-8604, Japan

* Corresponding authors:
Masahito Hayatsu, Institute for Agro-Environmental Sciences, National Agriculture and Food Research Organization (NARO), 3-1-3, Kannondai, Tsukuba, Ibaraki 305-8604, Japan. Phone and fax: 81-298-8309. e-mail: hayatsu@affrc.go.jp
Kiwamu Minamisawa, Graduate School of Life Sciences, Tohoku University, 2-1-1 Katahira, Aoba-ku, Sendai, Miyagi 980-8577, Japan. Phone and fax: 81-22-217-5684. e-mail: kiwamu@ige.tohoku.ac.jp

Methods

Culture of C110 and media used in field experiments

A mixture of 63 *nosZ+ Bradyrhizobium diazoefficiens* (C110) was cultured for five days at 30°C in flasks using modified AG medium[1] supplemented with 0.3 g L^{-1} arabinose, 0.3 g L^{-1} sodium gluconate, and 0.3 g L^{-1} yeast extract at 30°C, with rotational shaking at 120 rpm. Cells were harvested by centrifugation at 10,000 ×*g* for 15 min at 4°C. The cell pellets were washed three times with sterile water and were suspended at a concentration of 10^{10} cells mL^{-1} in sterilized water. Soybean seeds were inoculated with the cells at 10^{10} cells per seed. For the native rhizobia (control) treatment, 1 mL of soil suspension containing 0.1 g of the experimental field soil per seed was used as the inoculum.

Quantification of nodC *gene of* Bradyrhizobium diazoefficiens *in the experimental field or orchard soil*

DNA was extracted from 500 mg soil, using a FastDNA Spin kit for soil (Qbiogene, Vista, CA, USA) according to previous reports[2]. Extracted soil DNA was purified using an UltraClean 15 DNA purification kit (MO BIO Laboratories, Solana Beach, CA, USA). The population size of *Bradyrhizobium diazoefficiens* was determined by SYBR green real-time PCR using the StepOne real-time PCR system (Applied Biosystems, Carlsbad, CA, USA) targeting *nodC* gene with specific primer set previously reported[3]. Each 20 µL PCR reaction contained 10 µL of Takara SYBR® Premix Ex Taq™, 0.4 µL of Rox (Takara Bio Inc., Shiga, Japan), 0.2 µM of each primer, and 2 µL of standard or unknown soil DNA. Thermal cycling included an initial denaturation step for 2 min at 94°C followed by 40 cycles of amplification (30 s at 94°C, 30 s at 57°C, and 30 s at 72°C). The generation of specific PCR products was confirmed by means of melting-curve analysis.

Standardization of templates was performed with a dilution series (10^7−10^2 copies μL^{-1}) of the almost total length of *nodC* gene amplicons of *B. diazoefficiens* USDA110 generated with the primer set (nodCBjF and nodCBjR)[4].

Field experiment settings

The study sites for the field experiments were located at NIAES, Japan (36°01′N, 140°07′E). A *nosZ*− dominant Andosol field (*nosZ*− = 98%, *nosZ*+ = 2%) was divided into 6 m × 6 m plots. The experimental design was a randomized block design with 2 treatments × 5 replicates.

To increase the proportion of cotyledon emergence, soybean seeds were germinated in trays filled with moist vermiculite for one day. Then, soybean (*Glycine max* [L.] Merr., ver. Tachinagaha) seeds were planted in biodegradable Jiffy pots (Jiffy International AS, Kristiansand, Norway) filled with Andosol soil collected from an orchard located about 100 m away from the experimental field. The orchard soil was chosen because it has a lower native *B. diazoefficiens* population than, but soil properties similar to, the experimental field. Soybean seeds were inoculated with C110 (*nosZ*+) or 50 ml of soil from the experimental field (native) on June 26, 2013 and June 18, 2014, as described in the section ***Culture of C110 and media for field experiments***. Soybeans seedlings were then grown in a greenhouse under natural light and transplanted into the fields on July 3, 2013 and June 25, 2014. Basal fertilizer was applied as a compound fertilizer (30 kg N ha^{-1}) one day before transplanting. Seedlings were transplanted with their biodegradable pots. Basal fertilizer was applied as a compound fertilizer (30 kg N ha^{-1}, as ammonium-N, 50 kg ha^{-1}; P_2O_5-equivalent, 50 kg ha^{-1}; K_2O-equivalent) one day before transplanting of the soybean pots. Soybean crops were harvested on October 17, 2013 and October 10, 2014, the aboveground residues were removed, and only the roots and stubble were left in the field.

Nodule occupancy by **Bradyrhizobium diazoefficiens** *with* **nosZ** *gene*

To determine the number of nodules and their occupancy by the inoculants, we collected three roots from each plot. We divided the root samples into two parts (root segments growing inside versus outside the Jiffy pot) and separately determined nodule number and occupancy for these two categories of roots. The nodule occupancy by strains with different alleles of the *nosZ* gene was examined using eight nodules excised from each of the two categories of plant roots. Nodules were crushed using sterilized toothpicks and suspended in 40 μL of sterilized water. We then added 50 μL of BL buffer (40 mM Tris, 1 mM EDTA-Na, 1% Tween 20, 0.5% Nonidet P-40, pH 8.0) and 0.3 U of proteinase K (30 U mg−1) to the bacteroid cell suspension. Cell mixtures were incubated at 60°C for 20 min and then at 95°C for 5 min. After centrifugation (2800 ×g, 15 min., 20°C), the supernatants were used as PCR templates.

The *nosZ*-specific primer pair and previously reported[3] primer pairs specific to *nodC* of *B. diazoefficiens* or *B. elkanii* were used to detect *B. diazoefficiens nosZ*. For simultaneous detection of *nodC* of *B. diazoefficiens* and *nosZ*, two pairs of primers targeting them were used in a single reaction tube. Multiplex PCR was performed according to the manual of the TaKaRa multiplex PCR kit (TaKaRa, Shiga, Japan). The multiplex PCR reaction was performed in a total volume of 20 μL containing 10 μL of Mix 2, 0.1 μL of Mix 1, 1μL of DNA template, and 0.2 μM each primer. PCR reactions were performed on the GeneAmp® PCR System 9700 (Applied Biosystems, Foster City, CA, USA) with initial denaturation at 94°C for 1 min and 30 cycles of denaturation at 94°C for 30 s, annealing at 57°C for 90 s, and elongation at 72°C for 90 s. The PCR products were size separated on 4% NuSieve 3:1 agarose gel (Lonza, Rockland, ME, USA) and stained with ethidium bromide. An UV transilluminator and photograph system with an analyst computer program was employed for visualization. For samples showing no *nodC* band of *B. diazoefficiens*, another PCR was performed with primer pairs specific to *nodC* of *B. elkanii*. The PCR program was an initial step of 94°C for 2 min, followed by 30 cycles of 94°C for 30 s, 65°C for 30 s, and 72°C for 30 s. The PCR mixture (total volume of 20 μL) contained 2 μl of 10× buffer, 0.2 mM of each dNTP, 0.1 μl of ExTaq Hot Start Version (Takara), 1 μL of DNA template, and 0.2 μM of each primer.

Expression analysis of the* nirK *and* nosZ *gene

After the incubation experiments, nodules were quickly frozen in liquid nitrogen and stored at −80°C until analysis. Total RNA was extracted from 400 mg of nodules using the TRIzol reagent (Invitrogen, Carlsbad, CA, USA), following the manufacturer's protocol. RNA was treated with TurboDNase (Ambion, Austin, TX, USA) and purified using the RNeasy Mini Kit (Qiagen GmbH, Hilden, Germany). Total RNA was quantified by microspectrophotometry (Nano-Drop Technologies, Inc.). RNA integrity was measured using an Agilent 2100 Bioanalyzer (Agilent Technologies, Inc.). RNA with a RNA integrity number (RIN) above 7.0 was used for real-time qRT-PCR. The absence of contaminating genomic DNA after DNase treatment was verified by PCR analysis using previously reported[3] specific primers for the *sigA* genes.

Total RNA was subjected to qRT-PCR using a Prime-Script RT regent kit (TaKaRa). The RT reaction was performed in 20 µL reaction mixtures that contained 8 µL RNA, 4 µL of 5× PrimeScript® Buffer (for Real Time analysis), 1 µL of PrimeScript® RT Enzyme Mix I, 1 µL of random 6-mers (100 µM), and 5 µL of ddH_2O (TaKaRa). The RT reaction protocol included incubation at 37°C for 15 min and 85°C for 5 s. The qPCR was performed in Step One plus (Applied Biosystems) using SYBR Premix Ex TaqII (TaKaRa Biotechnology) and specific primers for the *nosZ* (nosZ-1f and nosZ-1r)[3], *nirK* (B_nirK-f, 5′-ATGGCGACTATGTCTGGGAG-3′; B_nirK-r, 5′-CGTGACATAGGCGTAGATGC-3′) and *sigA* genes.

Each 20 µL PCR reaction contained 10 µL of Takara SYBR® Premix Ex Taq™ II, 0.4 µL of Rox (Takara Bio Inc., Shiga, Japan), 0.2 µM of each primer and 2 µL of standard or unknown DNA. Thermal cycling included an initial denaturation step for 2 min at 94°C followed by 40 cycles of amplification (15 s at 94°C, 30 s at 55°C, and 30 s at 72°C). The generation of specific PCR products was confirmed by means of melting-curve analysis. Standardization of templates for each gene was performed with a dilution series (10^7−10^2 copies µL^{-1}) of *nosZ*, *nirK*, and *sigA* gene amplicons of *B. diazoefficiens* USDA110 generated with the same qPCR primers.

Measurements of N_2O flux

N_2O emission was measured every two days during the cultivation and nodule decomposition period (from July 2, 2013 to January 26, 2014 and June 7, 2014 to December 26, 2014) and every four days during the remainder of the period using automated gas sampling systems with automatically closing chambers (90 × 90 cm; 60 cm or 110 cm in height, depending on plant height)[5]. Each chamber was closed for 1 h starting at 16:00 h on the measurement day. We collected gas samples in evacuated 15 mL vials from the atmosphere inside each chamber at 0, 30, and 60 min after closure of the chamber. Sampling was performed from 16:00 to 17:00 h to obtain the daily average flux, and the timing was adapted from a previous study of diel changes in the N_2O flux in a nearby field[6]. The gas samples were then analyzed to determine the N_2O concentration using a gas chromatography equipped with a CH_4-doped ^{63}Ni electron capture detector at 340°C (GC-2014, Shimadzu, Kyoto, Japan) using a headspace auto-sampler (HS-2B, Shimadzu). The headspace auto-sampler was modified for trace gas analysis by replacement of the original syringe with a 2 mL gas-tight syringe (Pressure-Lok series A, VICI Precision Sampling, Baton Rouge, LA, USA) and by removal of the original heating unit. N_2 was used as the carrier gas. N_2O emission was calculated from the rate of increase in N_2O concentration between the three measured concentrations. Standard gas (0.3, 0.5, 1, 2.5, and 5 ppm N_2O) were analyzed before and after daily analyses of the samples. The coefficients of variation for repeated analyses of the standard gas (N_2O, 0.5 ppm) were 0.48% for N_2O (n = 40). The fluxes were calculated from the linear increase or decrease of the gas concentrations during the sampling period.

Bulk soil sampling and mineral nitrogen measurements

Soil samples were taken periodically in triplicate. Surface soil (0 to 5 cm) was randomly collected from five points in each plot and mixed together in a plastic bag to produce a composite sample. The bulk soil samples were immediately transferred to the laboratory for analysis. Samples of fresh soil (10 g) were extracted with 100 mL of KCl solution (100 g KCl L^{-1}). We used the copper-cadmium reduction and diazotization method to determine the NO_3^- content and used the indophenol blue method to analyze the NH_4^+ content using a TRRACS continuous-flow analyzer (Bran+Luebbe,

Norderstedt, Germany).

Rhizosphere soil, root, and nodule mineral nitrogen measurement

After the incubation experiment for N_2O production rates (Methods in the main text), rhizosphere soil, root, and nodule samples were extracted with KCl solution (1:10 ratio; 100 g KCl L^{-1}). NO_3^- and NH_4^+ contents were measured using with a TRRACS continuous-flow analyzer (Bran+Luebbe, Norderstedt, Germany).

Statistical analysis

All statistical analysis was conducted using version 18.0 of the PASW statistics software (SPSS Inc., New York, NY, USA). N_2O production rate and the proportion of nodule occupancy by the inoculants were evaluated using *t*-tests (two-sided). Normal distribution was assumed. The homogeneity of varience was tested by Levene's test. The effect of *nosZ*+ on N_2O emission based on two years of field data was evaluated using a mixed linear model.

Table S1. *Bradyrhizobium diazoefficiens* strains and cells mixture used in this study.

Strains and mixture	ITS accession number	Source*	Reference
Bradyrhizobium diazoefficiens strains			
HK1-31	AB983868	Hokkaido (Fluvisol)	Shiina et al. 2014#
HK1-32	AB983869	Hokkaido (Fluvisol)	Shiina et al. 2014
HK2-8	AB983876	Hokkaido (Fluvisol)	Shiina et al. 2014
HK3-7	AB983881	Hokkaido (Fluvisol)	Shiina et al. 2014
HK3-13	AB983884	Hokkaido (Fluvisol)	Shiina et al. 2014
HK3-21	AB983885	Hokkaido (Fluvisol)	Shiina et al. 2014
HK3-25	AB983886	Hokkaido (Fluvisol)	Shiina et al. 2014
KS1-16	AB983952	Miyagi (Gleysol)	Shiina et al. 2014
KS2-2	AB983956	Miyagi (Gleysol)	Shiina et al. 2014
KS2-3	AB983957	Miyagi (Gleysol)	Shiina et al. 2014
YM1-1	AB983962	Yamagata (Gleysol)	Shiina et al. 2014
YM1-3	AB983964	Yamagata (Gleysol)	Shiina et al. 2014
YM1-4	AB983965	Yamagata (Gleysol)	Shiina et al. 2014
YM1-5	AB983966	Yamagata (Gleysol)	Shiina et al. 2014
YM1-6	AB983967	Yamagata (Gleysol)	Shiina et al. 2014
YM1-7	AB983968	Yamagata (Gleysol)	Shiina et al. 2014
YM2-2	AB983972	Yamagata (Gleysol)	Shiina et al. 2014
YM2-3	AB983973	Yamagata (Gleysol)	Shiina et al. 2014
YM2-5	AB983974	Yamagata (Gleysol)	Shiina et al. 2014
YM2-7	AB983976	Yamagata (Gleysol)	Shiina et al. 2014
YM3-2	AB983980	Yamagata (Gleysol)	Shiina et al. 2014
YM3-4	AB983981	Yamagata (Gleysol)	Shiina et al. 2014
NG1	AB983989	Niigata (Gleysol)	Shiina et al. 2014
NG2	AB983990	Niigata (Gleysol)	Shiina et al. 2014
NG5	AB983993	Niigata (Gleysol)	Shiina et al. 2014
TS1-1	AB983995	Ibaraki (Gleysol)	Shiina et al. 2014
TS1-2	AB983996	Ibaraki (Gleysol)	Shiina et al. 2014
TS1-3	AB983997	Ibaraki (Gleysol)	Shiina et al. 2014
TS1-4	AB983998	Ibaraki (Gleysol)	Shiina et al. 2014
TS1-5	AB983999	Ibaraki (Gleysol)	Shiina et al. 2014
TS1-6	AB984000	Ibaraki (Gleysol)	Shiina et al. 2014
TS2-1	AB984001	Ibaraki (Gleysol)	Shiina et al. 2014
TS2-10	AB984005	Ibaraki (Gleysol)	Shiina et al. 2014
TS3-4	AB984007	Ibaraki (Gleysol)	Shiina et al. 2014
TS3-5	AB984008	Ibaraki (Gleysol)	Shiina et al. 2014
TS3-6	AB984009	Ibaraki (Gleysol)	Shiina et al. 2014
TS3-7	AB984010	Ibaraki (Gleysol)	Shiina et al. 2014
TS3-19	AB984015	Ibaraki (Gleysol)	Shiina et al. 2014
TS4-125	AB984019	Ibaraki (Andosol)	Shiina et al. 2014

FK2-2	AB984034	Fukuoka (Gleysol)	Shiina et al. 2014
FK2-3	AB984035	Fukuoka (Gleysol)	Shiina et al. 2014
FK2-4	AB984036	Fukuoka (Gleysol)	Shiina et al. 2014
FK2-6	AB984038	Fukuoka (Gleysol)	Shiina et al. 2014
KM1-1	AB984045	Kumamoto (Gleysol)	Shiina et al. 2014
KM1-2	AB984046	Kumamoto (Gleysol)	Shiina et al. 2014
KM1-3	AB984047	Kumamoto (Gleysol)	Shiina et al. 2014
KM1-5	AB984049	Kumamoto (Gleysol)	Shiina et al. 2014
KM1-6	AB984050	Kumamoto (Gleysol)	Shiina et al. 2014
KM2-5	AB984054	Kumamoto (Gleysol)	Shiina et al. 2014
KM2-6	AB984055	Kumamoto (Gleysol)	Shiina et al. 2014
KM2-7	AB984056	Kumamoto (Gleysol)	Shiina et al. 2014
KM2-8	AB984057	Kumamoto (Gleysol)	Shiina et al. 2014
KM4-1	AB984069	Kumamoto (Gleysol)	Shiina et al. 2014
KM4-2	AB984070	Kumamoto (Gleysol)	Shiina et al. 2014
MY1-2	AB984078	Miyazaki (Gleysol)	Shiina et al. 2014
MY1-3	AB984079	Miyazaki (Gleysol)	Shiina et al. 2014
MY1-4	AB984080	Miyazaki (Gleysol)	Shiina et al. 2014
KG1-5	AB984092	Kagoshima (Gleysol)	Shiina et al. 2014
KG1-11	AB984097	Kagoshima (Gleysol)	Shiina et al. 2014
KG1-13	AB984098	Kagoshima (Gleysol)	Shiina et al. 2014
KG2-5	AB984100	Kagoshima (Andosol)	Shiina et al. 2014
KG2-6	AB984101	Kagoshima (Andosol)	Shiina et al. 2014
KG2-73	LC101818	Kagoshima (Andosol)	Shiina et al. 2014
Bradyrhizobium diazoefficiens cells mixture			
C110	-	Cells mixture of 63 *B. diazoefficiens* strains	This study

* Source of *B. diazoefficiens* strains indicates isolated prefecture in Japan. The word in parentheses indicates soil types of isolated fields.
# Shiina et al. 2014 (ref.7)

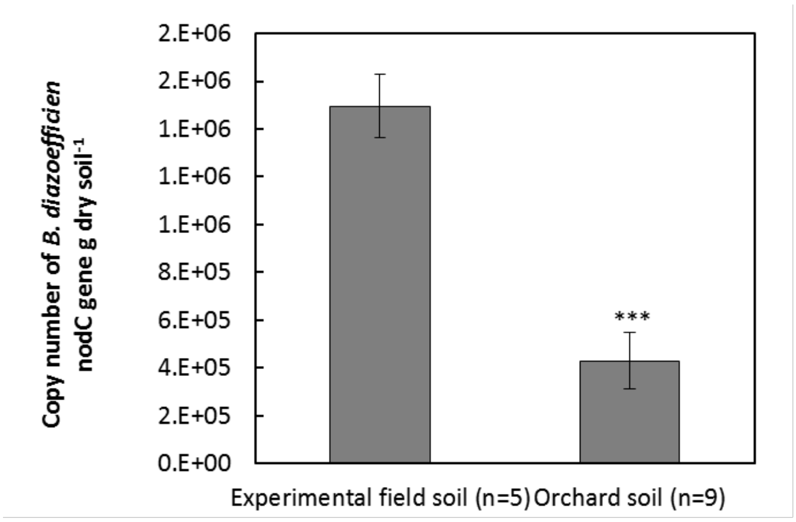

Supplemental Fig. S1. nodC copy numbers in the Andosol experimental field soil and the nearby Andosol orchard soil that was used for soybean seedling pots.
Values are means ± SD.
*** $P < 0.001$

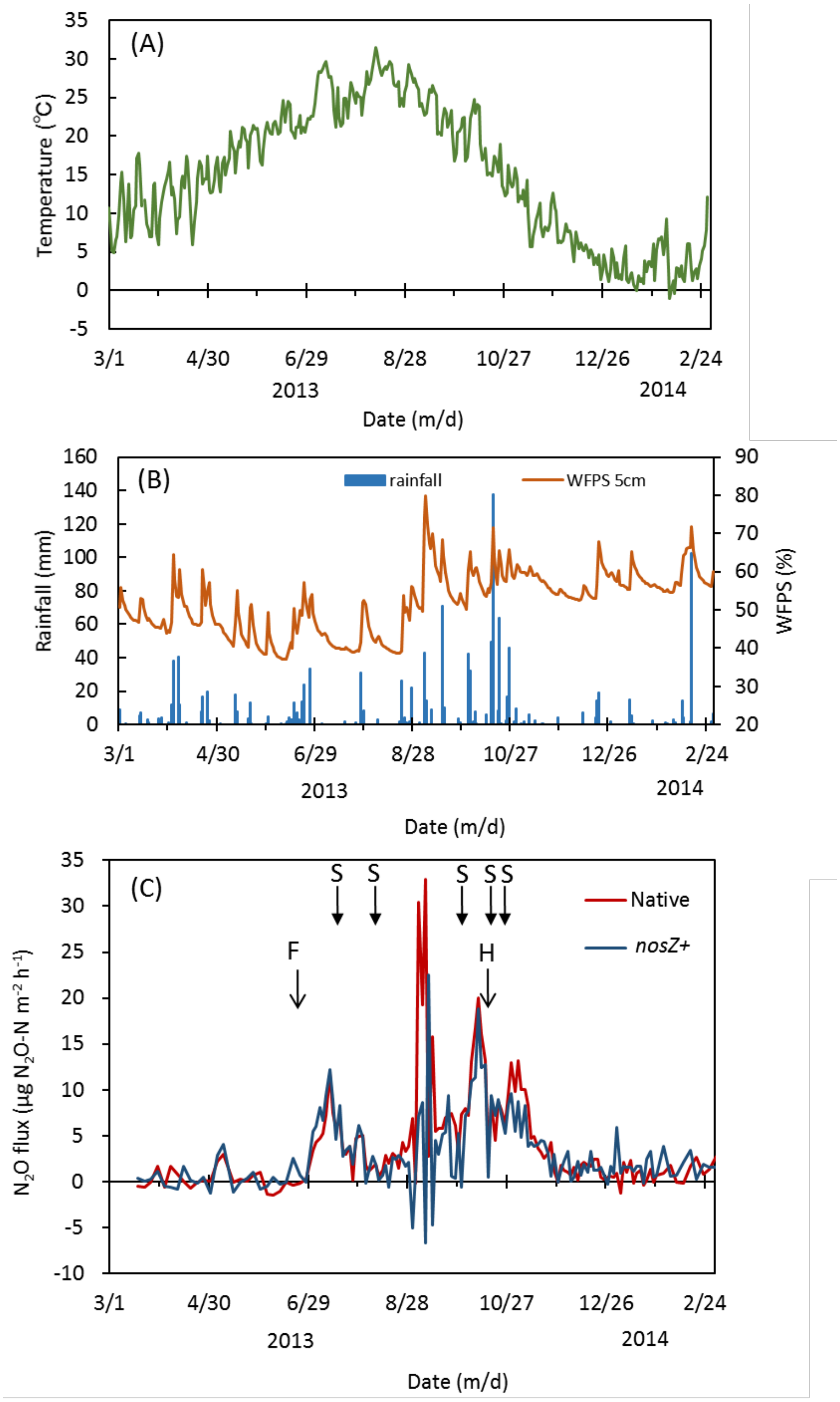

Supplementary Fig. S2. Seasonal changes of (A) air temperature, (B) rainfall and water-filled pore space (WFPS) at a depth of 5 cm, and (C) N_2O fluxes from an Andosol field in the 2013 experiment. Soybean seeds were inoculated at sowing with a mixed culture of *B. diazoefficiens* strains (*nosZ*+) or native strains (Native; *nosZ*− dominant) (n = 5). F, fertilizer application; H, harvesting of soybean; S: sampling for N_2O production rates of soil, roots, and nodules.

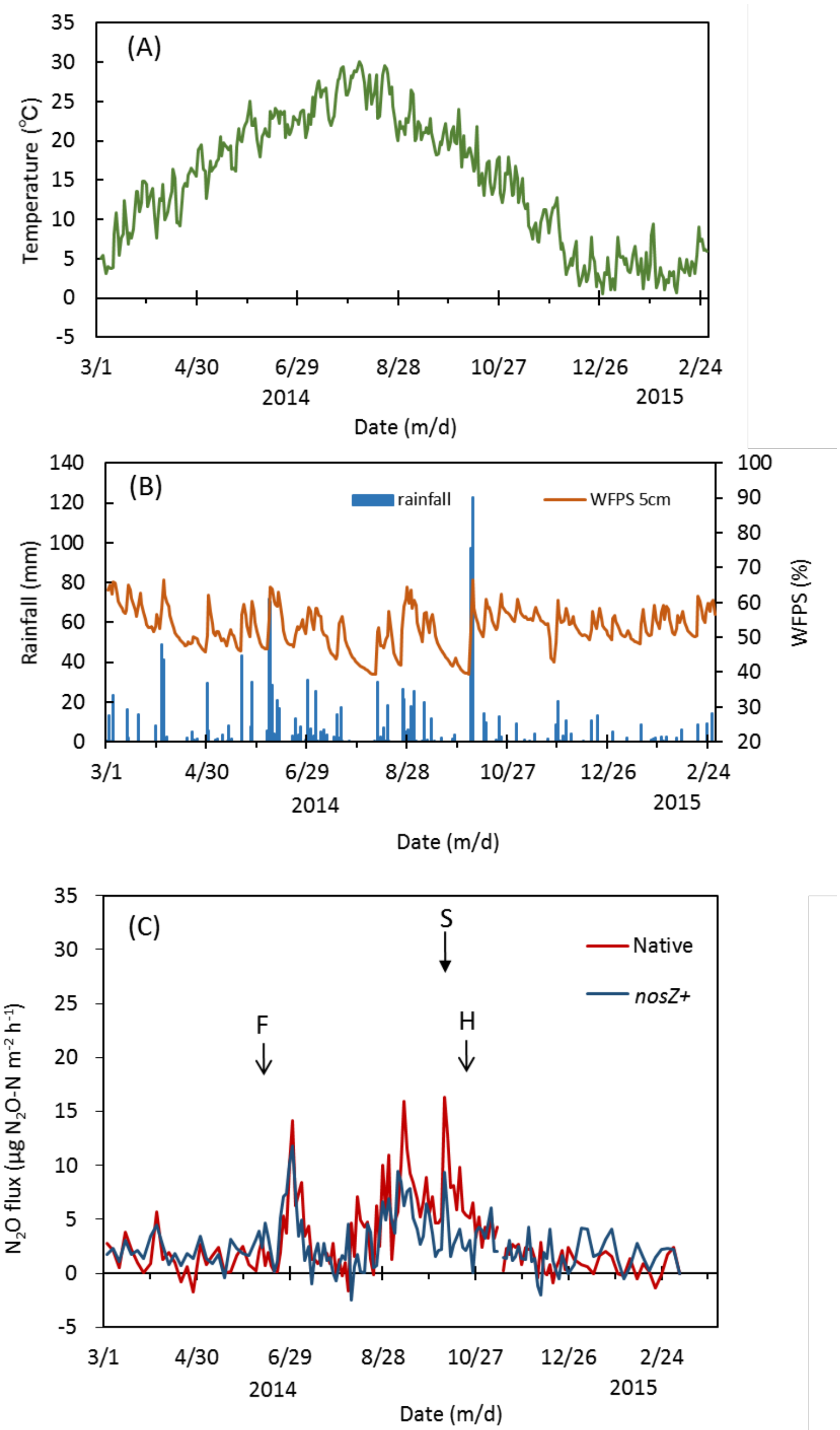

Supplementary Fig. S3. Seasonal changes of (A) air temperature, (B) rainfall and water-filled pore space (WFPS) at a depth of 5 cm, and (C) N_2O fluxes from an Andosol field in the 2014 experiment. Soybean seeds were inoculated at sowing with a mixed culture of *B. diazoefficiens* strains (*nosZ*+) or native strains (Native; *nosZ*− dominant) (n = 5). F, fertilizer application; H, harvesting of soybean; S: sampling for N_2O production rates of soil, roots, and nodules.

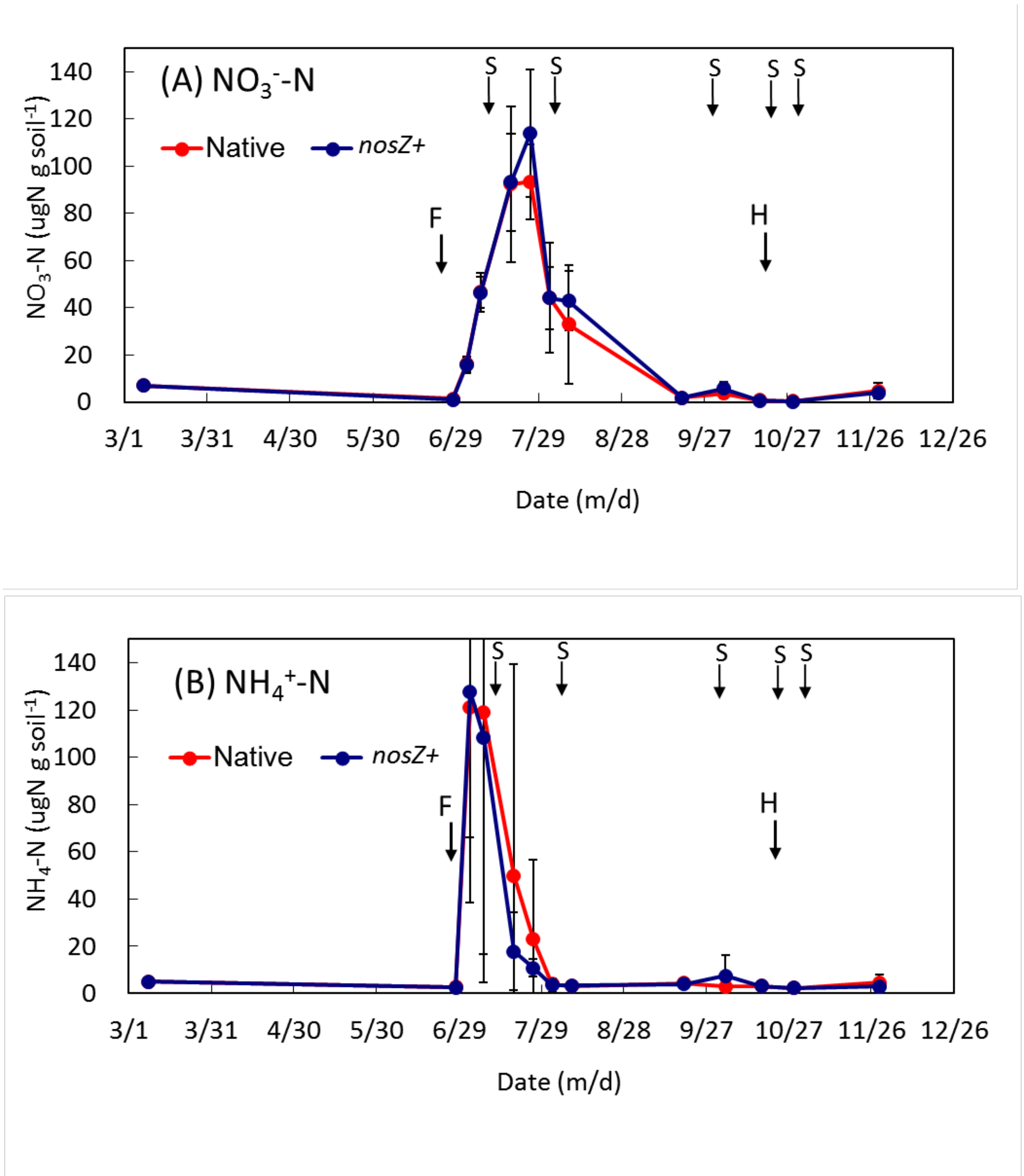

Supplementary Fig. S4. Seasonal changes of soil NO_3^- (A) and NH_4^+ (B) contents during the field experiment in 2013. Soybean seeds were inoculated at sowing with a mixed culture of *B. diazoefficiens* strains (*nosZ*+) or native strains (Native; *nosZ*− dominant). F, fertilizer application; H, soybean harvest: S: sampling for N_2O production rates of soil, roots, and nodules. Values are means ± SD (*n* = 5).

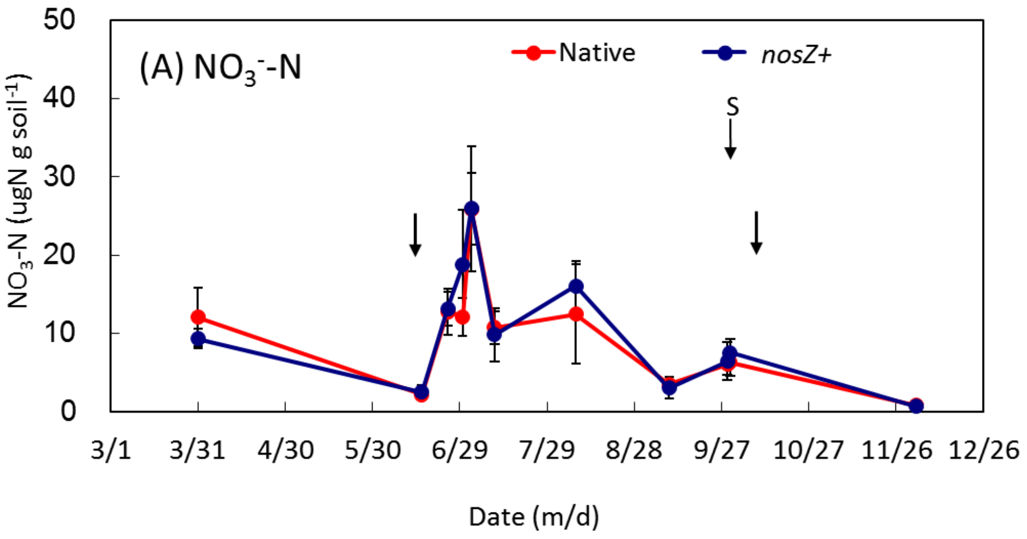

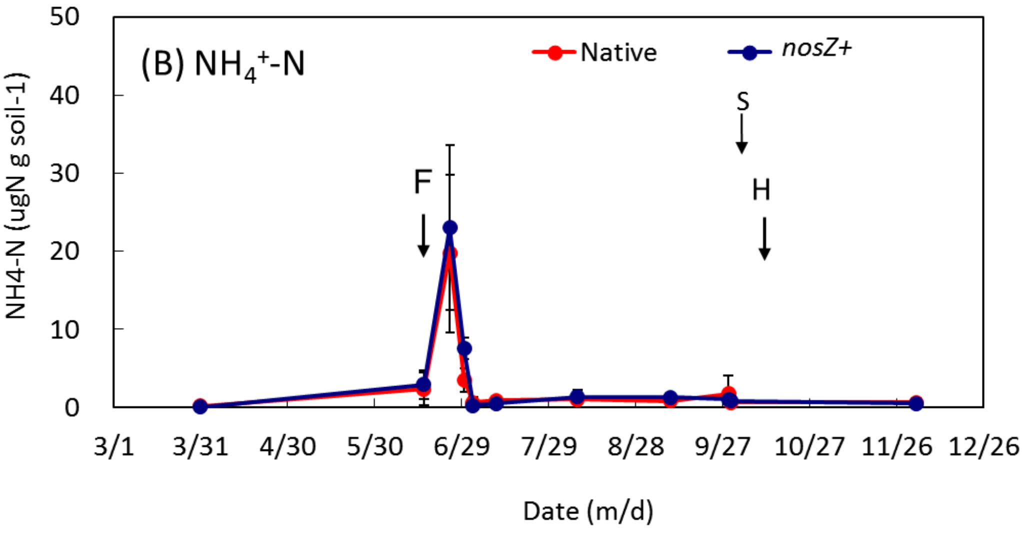

Supplementary Fig. S5. Seasonal changes of soil (A) NO_3^- and (B) NH_4^+ content in the field experiment in 2014. Soybean seeds were inoculated at sowing with a mixed culture of *B. diazoefficiens* strains (*nosZ*+) or native strains (Native; *nosZ*− dominant). F, fertilizer application; H, soybean harvest; S: sampling for N_2O production rates of soil, roots, and nodules. Values are means ± SD (n = 5).

References

1. Sadowsky M. J., Tully R. E., Cregan P. B. & Keyser H. H. Genetic diversity in *Bradyrhizobium japonicaum* serogroup 123 and its relation to genotype-specific nodulation of soybean. *Appl. Environ. Microbiol*. **53**, 2624-2630 (1987).
2. Hoshino, Y. T. & Matsumoto, N. An Improved DNA Extraction Method Using Skim Milk from Soils That Strongly Adsorb DNA. *Microbes Environ*. **19**, 13-19 (2004).
3. Itakura, M. et al., Mitigation of nitrous oxide emissions from soils by Bradyrhizobium japonicum inoculation, *Nature Clim. Change* **3**, 208-212 (2013).
4. Barcellos, F. G. *et al*. Evidence of Horizontal Transfer of Symbiotic Genes from a *Bradyrhizobium japonicum* Inoculant Strain to Indigenous Diazotrophs *Sinorhizobium* (*Ensifer*) *fredii* and *Bradyrhizobium elkanii* in a Brazilian Savannah Soil. *Appl. Environ. Microbiol.* **73**, 2635–2643 (2007).
5. Akiyama, H. *et al.* Automated sampling system for long-term monitoring of nitrous oxide and methane fluxes from soils. *Soil Sci. Plant Nutri*. **55**, 435-440 (2009).
6. Akiyama H. & Tsuruta H. Effect of organic matter application on N_2O, NO, and NO_2 fluxes from an Andisol field, Global Biogeochemical Cycles, 17, DOI: 10.1029/2002GB002016 (2003).
7. Shiina Y. *et al*. Correlation between soil type and N_2O reductase genotype (*nosZ*) of indigenous soybean bradyrhizobia: *nosZ*-minus populations are dominant in Andosols. *Microbes Environ*. **29**, 420-426
